# A Randomized Controlled Trial Investigating the Effects of Undulatory, Staggered, and Linear Load Manipulations in Aerobic Training on Oxygen Supply, Muscle Injury, and Metabolism in Male Recreational Runners

**DOI:** 10.1186/s40798-019-0200-5

**Published:** 2019-07-22

**Authors:** Paulo Costa, Roberto Simão, Anselmo Perez, Maurício Gama, Rogério Lanchtermacher, Reinaldo Musialowski, Fábio Braga, Valeria de Mello Coelho, Alexandre Palma

**Affiliations:** 1 School of Physical Education and Sports, Rio de Janeiro Federal University, Av. Carlos Chagas Filho, 540 - Cidade Universitária, Rio de Janeiro, RJ 21940-901 Brazil; 2 Laboratory of Immunophysiology, Institute of Biomedical Sciences, Health Sciences Center, Federal University of Rio de Janeiro, Rio de Janeiro, Brazil; 3 Laboratory of Exercise Physiology, Federal University of Espirito Santo, Vitoria, ES Brazil

**Keywords:** Aerobic training, Taper, load manipulation, aerobic fitness, Oxygen supply, Muscle injury

## Abstract

**Background:**

Taper is considered as a strategy to avoid overtraining and increase peak performance in athletes. Because aerobic exercise increases the level and duration of independence during the lifespan, the participation of recreational athletes has increased in running events around the world. However, the effects of using load reduction in their training and aerobic performance are still not well known.

**Objectives:**

Our goal was to compare load manipulations, using tapering-like periods in the end of recreational athletes training evaluating alterations in oxygen supply, muscle injury, and metabolism markers.

**Methods:**

Healthy males (*n* = 88, 20–35 years old) were randomly distributed in groups using a combination of two mesocycles of 4 weeks, undulatory and staggered, with two daily microcycles, undulatory and linear. Undulatory-undulatory (Und-Und) and undulatory-linear (Und-Lin) groups had load reduction in the final week of training while the staggered-undulatory (Sta-Und) and staggered-linear (Sta-Lin) groups did not. Cardiorespiratory capacity (V̇O_2max_), body mass index (BMI), and body fat % were evaluated. Blood samples were also collected to analyze hematocrit (Ht), mean corpuscular hemoglobin (MCHC), circulating levels of hemoglobin (Hb), creatine kinase (CK), lactate dehydrogenase (LDH), aspartate aminotransferase (AST), alanine aminotransferase (ALT), urea (U), cortisol (C), free testosterone (FT), and free T/C ratio.

**Results:**

After 8 weeks of training, Und-Und had the highest trend to increase V̇O_2max_. Both Und-Und and Sta-Lin reduced body fat %. Und-Und showed a significant increase in MCHC, T and Free T/C ratio, a positive trend to increase Ht% and Hb levels, and a trend to decrease CK, LDH, and C circulating levels. Sta-Lin presented a trend to decrease Ht% and Hb levels, a significant increase in CK, LDH, AST, ALT circulating levels, and a decrease in Free T/C ratio.

**Conclusion:**

The use of daily undulatory training load provides better gains for aerobic fitness while the use of staggered load, mainly associated with linear load, promotes inadequate recovery in recreational runners.

## Key Points

- The use of taper-like periods after undulatory training volume loads can contribute to increase cardiorespiratory fitness and to benefit recovery while linear loads promote inadequate recovery in recreational runners.
- Recreational runners that perform undulatory cycles weekly and monthly with a decrease in volumes after 4 weeks of training increase their cardiorespiratory fitness and free testosterone/cortisol ratio and decrease the circulating levels of muscle injury markers after 8 weeks of training.
- Recreational runners that perform weekly linear loads and monthly staggered loads, without a taper-like period after the training, increase serum markers of protein catabolism and muscle injury and decrease free testosterone/cortisol ratio, indicating inefficient recovery after eight weeks of training.

## Background

Intensive exercise or inadequate recovery causes overtraining, which correspond to a state of prolonged fatigue and consequently decreased physical performance [1–4]. Overtraining promotes several psychological symptoms such as fear of competition, general apathy, emotional stress, and depression [5, 6], which can lead to higher susceptibility to immunodeficiency and infections [1, 5]. Moreover, overtraining causes physiological alterations including reduction of muscle glycogen [7] and in the circulating levels of catecholamines [2], lactate [2, 8], and free testosterone (FT) [1, 2, 9, 10]. On the other hand, it increases the circulating levels of urea (U) [2, 9, 10], creatine kinase (CK) [4, 11], oxidative enzymes [12], and cortisol (C) [2, 9, 11, 13]. Excepting one study showing a decrease in the levels of lactate dehydrogenase (LDH) [14], other groups have shown an increase in the levels of this enzyme, when taper was used [8, 15]. Overtraining leads to endocrine disorders such as a decrease of more than 30% in the FT/C ratio [2, 10, 16]. Initially, the increased levels of cortisol could compromise training adaptation because it contributes to decrease glycogen reserve and neuromuscular activity [17]. It is also described that short-endurance exercise, chronic fatigue, and insufficient recovery modulate hypothalamic–pituitary–adrenal axis increasing adrenocorticotrophic hormone/cortisol ratio due to decreased sensitivity of the pituitary [18]. The adaptive response to the HPA imbalance can promote a reduction in the cortisol activity thus regulating metabolic actions, increasing gluconeogenesis, or central action, altering adrenoreceptors sensitivity influencing on analgesia, for instance [18, 19]. In overtraining, an efficient strategy to optimize the performance avoiding or reversing the deleterious effects and alteration in the neuroendocrine system is to promote supercompensation through adequate recovery [20, 21]. In this regard, the law of overcompensation refers to adequate recovery after various training loads and/or a training session in order to achieve better skill levels [22, 23]. High-level athletes always possess the characteristic of accumulations of training hours that involve the manipulation of volume, intensity, frequency, duration of the session, and type of exercise. In this way, recovery from accumulation of training loads becomes necessary to obtain positive effects. Several studies show that reduction of aerobic training loads prior to competition, also known as taper, has been considered by coaches to avoid detraining [24–28], overtraining [25, 29, 30], and to optimize performance of athletes [2, 9, 28–30]. Indeed, taper is a common strategy among competitive athletes to ensure proper recovery [28, 31, 32].

During the reduction of aerobic training, several variables can be manipulated and even combined in an attempt to maximize performance. Some of these variables include the weekly reductions in frequency, volume, intensity, duration, and type of taper [33–37]. Models of taper include step, exponential with low or high decay, linear [25, 31], or through phases [35, 36]. Significant improvements have also been reported using undulatory and non-undulatory models of low training loads in runners [32, 33], swimmers [37, 38], cyclists [39, 40], and triathletes [5]. In this context, the application of training workload manipulation promotes a balance among distinct training loads and recovery periods, thus decreasing the chances of low performance due to injury, detraining, and overtraining [41, 42]. In relation to the use of aerobic training load reduction prior to competition, nine middle-distance runners were evaluated, being five submitted to high frequency taper and four to moderate frequency taper. Athletes showed an improvement in their performance during a 6-day taper. However, when runners rested every third day of the training, no significant gain was observed. The authors also tested levels of testosterone and cortisol and observed that hormonal levels propitious to anabolic processes were associated with an optimum performance [32]. Another group evaluated individually long-term training of 11 elite skiers and biathletes that had exceptional performances [43]. They also used a taper strategy. However, only three of 11 athletes had a resting day in the five final days prior to the peak performance while the majority of the elite athletes had rest days from training in the middle period of the peaking phase between the final 12 to 6 days prior the peak performance. Authors did not analyze markers for muscle injury or metabolic markers, such as testosterone and cortisol to correlate the athletes’ performance with their physiological conditions [43].

Regular physical exercise and fitness have been associated with reduction of mortality and improvement of health [44]. Aspects of health-related physical fitness include cardiorespiratory fitness, muscular strength, muscular endurance, body composition, and flexibility [45]. In this context, participation of recreational runners, also known as amateurs runners, has increased in running events around the world, including those of 5 or 10 km races, half-marathons, and marathons [45, 46]. Recreational runners have shown interest in training programs aiming their success to complete the running or to improve their run time. In this regard, inappropriate training can contribute for muscle stress and lesions or overtraining [46–48]. For this reason, studies of intensity manipulation have been performed also with recreational runners [49–51]. Models of load reduction in recreational athletes have also been proposed in runners [51–54] in order to observe hemodynamic variables and cardiorespiratory fitness. The use of taper in the training of recreational runners has been investigated and considered positive to improve physical adaptation [51]. In another study, sedentary individuals—who had not performed regular aerobic exercises during a previous year—were recruited, and entered into a 5-month training program, four to six times a week, with a 75% intensity of maximal oxygen uptake (V̇O_2max_) [55]. Other group also showed that sedentary individuals participated in a training program (30 min daily, 5 days/week) for 12 weeks to assess cardiorespiratory fitness [56]. In the current study, we considered the use of taper-like models as a possible strategy to optimize the cardiorespiratory fitness of recreational athletes in running events, based on several studies that show positive results of using taper in training of athletes [3, 25, 30–32, 34–36, 39].

Using a novel model of analysis, where tapering and distinct periodization were applied in the training program of recreational athletes submitted to aerobic exercise, we have shown that loading manipulation can ameliorate their aerobic fitness [57]. However, the physiological alterations in recreational athletes submitted to aerobic training programs with distinct load manipulation as well as reduction in the final period of exercise (taper-like period) are still unknown. In this study, we analyzed the cardiorespiratory capacity (V̇O_2max_) of recreational before and after 8 weeks of running, comparing the training effects with undulatory and/or non-undulatory load manipulations. We also evaluated the circulating levels of hematological, biochemical, and hormonal markers before, during, and after the aerobic training.

## Methods

### Subjects

The study recruited 97 individuals. However, 93 participants started training and 88 completed. All volunteers were informed verbally and in writing regarding the purpose, procedures, and potential risks of the study, according to the Helsinki declaration. Each participant provided written informed consent to be part in the study reviewed and approved under the protocol number 024/10 by the local Committee on Human Research in the University Hospital Clementino Fraga Filho at Federal University of Rio de Janeiro.

### Research Design

The inclusion criteria adopted in this study were males, in the age of 20 to 35 years; that frequently run at least twice a week, lasting between 30 and 120 min per day for at least 6 months; and agreed to not participate in any additional exercise during the study period. All participants were physically active and did not take part in any form of organized competitive athletics. The exclusion criteria included medical condition that could endanger their health, such as hypertension or cardiac abnormalities; tobacco use; functional limitations that could compromise their exercise capacity, such as orthopedic and neurological disorders; use of medication, either for treatment of an illness or ergogenic effect; positive response to the PAR-Q questionnaire; V̇O_2max_ lower than 33 ml kg^−1^ min^−1^; and body fat percentage greater than 25%. We should point out evidences linking low levels of cardiorespiratory fitness with increased cardiovascular mortality considering that the relative risk of death may be related to a lower functional capacity independently of the risk factors involved [58–62]. In this regard, V̇O_2max_ below 33 ml/kg/min has been classified as “Weak” [63] or “very poor” [64], and body fat % ≥ 25% was considered “weak” or “poor” and associated with cardiovascular risk [64, 65]. Diet was not prescribed but participants were recommended to eat healthy.

**Fig. 1:**
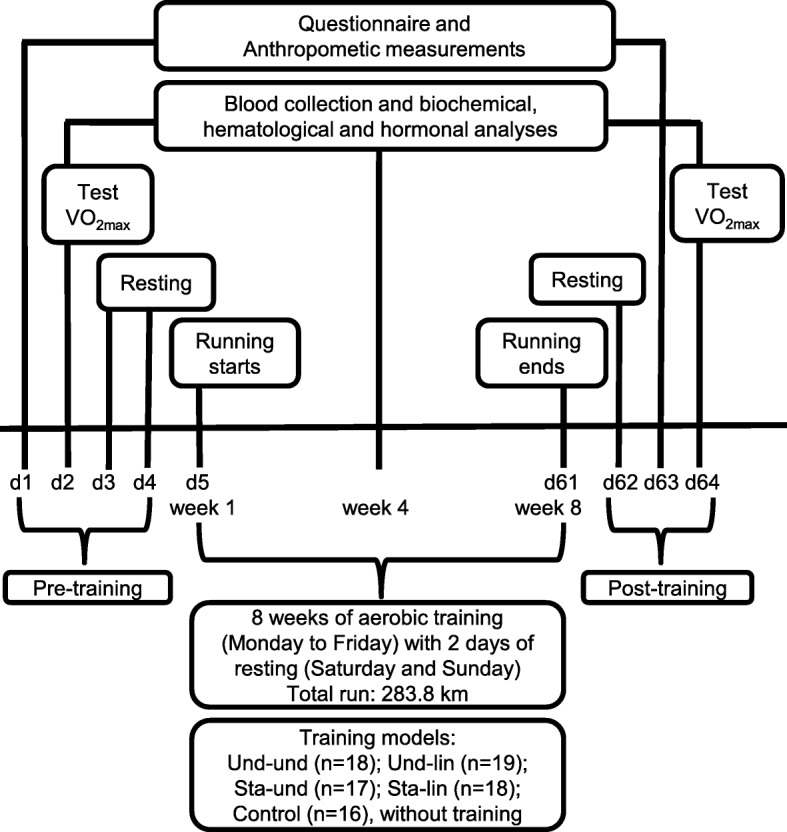
Experimental design of the study. Healthy males (*n* = 88, 20–35 years old) were submitted to physical and physiological evaluation before (pre-training), during (aerobic training), and after 8 weeks of aerobic training (post-training), as indicated. The participants performed 8 weeks of aerobic training randomly assigned into four groups based on distinct periodization models: undulatory-undulatory (Und-Und); undulatory-linear (Und-Lin); staggered-undulatory (Sta-Und); staggered-linear (Sta-Lin). Each training participant ran a total of 283.8 km throughout the distinct trainings. Control group maintained their usual aerobic training twice a week

**Fig. 2:**
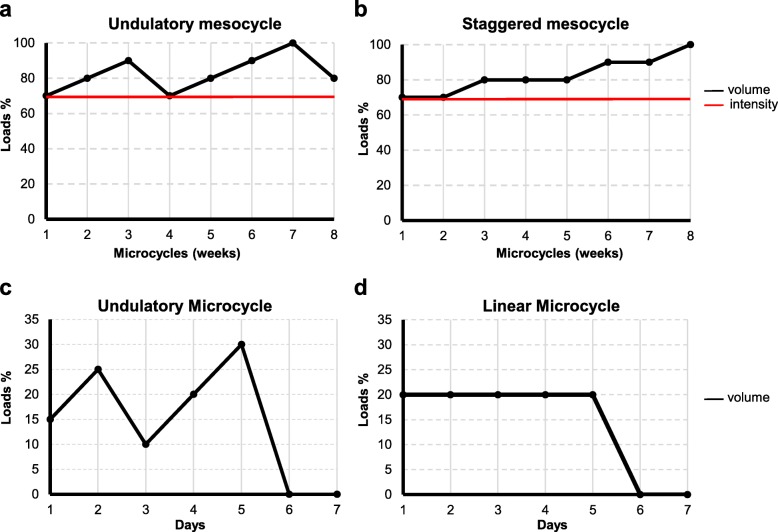
Load manipulation models applied to recreational athletes aerobic trainings. Participants were randomly distributed in training groups using a combination of two mesocycles of 4 weeks, **a** undulatory and **b** staggered, with two daily microcycles, **c** undulatory and **d** linear. Training intensity was maintained at 70% of V̇O_2max_ through changes in running speed. Each training group performed continuous running 5 days/week**.** Training intensity (% of V̇O_2max_ = 70) and volume (% of the maximum distance run/per week) are indicated in the graphs. Adapted from [5]

All participants answered a questionnaire that allowed verifying their usual training frequency (2 to 6 days per week, being 15.91%, 2 days; 37.51%, 3 days; 22.72%, 4 days; 18.18%, 5 days and 5.68% that run 6 days per week), weekly volume (14.4 to 55 km), and intensity (6.9 to 10 km/h). Participants were then submitted to anthropometric measurements, blood collection, and cardiorespiratory physical fitness in the pre-training period (Fig. 1). In these groups, the weekly mesocycle, which consists of 4 weeks, was manipulated in two ways, called “undulatory” and “staggered”, as previously described [57]. The daily microcycle (from Monday to Friday) was established also in two ways, called “undulatory” and “linear” [57]. All experimental groups performed the same overall training load, although the loads were distributed differently. The participants, randomly assigned into four groups, performed 8 weeks of aerobic training (Fig. 2). Two groups of trainings that used taper-like periods, undulatory-undulatory (Und-Und) (*n* = 18) and undulatory-linear (Und-Lin) (*n* = 19), had load reduction at two moments: after 4 and 8 weeks of training. The two other groups, staggered-undulatory (Sta-Und) (*n* = 17) and staggered-linear (Sta-Lin) (*n* = 18), did not used taper-like periods; there was first an increase in the load and no load reduction in the final week of training. The participants in these groups did not reach their final stage since they were monitored to maintain 70% of their V̇O_2max_ during the aerobic training. The control group included participants that usually train at least 2 days a week and maintained their usual routine during the 8 weeks of aerobic training period of the study (*n* = 16). Each training group performed continuous running 5 days/week, and had three physical education professionals monitoring and controlling the training load.

The aerobic training was performed between the months of September and November, spring season in Rio de Janeiro. For 8 weeks, 5 days per week, participants started training around 8:00 am on a 400 m official track of athletics (outdoors), with markings every 10 m. Each participant received a digital clock with a stopwatch in order to control their own time and pace. In addition, two researchers were positioned in the markings 200 m and 400 m to supervise the pace of the runners. The intensity for every participant was determined by their V̇O_2max_ of 70% and controlled by the speed of the running (range 3 to 10.6 km per session; 26.07 to 91.62 min/session), with a weekly volume between 30.1 and 43 km and total volume (100%) of 283.3 km, at the end of 8 weeks, which in average corresponded to 7.095 km/day. Each participant received the distance and total execution time daily, based on their V̇O_2max_. Studies have reported the use of % of V̇O_2max_ as a parameter for the prescription of exercise intensity in order to improve cardiorespiratory fitness [66–69].

The randomized distribution of volunteers in the different groups was done according to their V̇O_2max_. The V̇O_2max_ test was measured by one of the authors of the study. Another author made the ranking decreasing the V̇O_2max_ values and distributing volunteers in each groups. For instance, higher value of V̇O_2max_: Und-Und group; second higher value: Und-Lin group; third higher value: Sta-Und group; fourth higher value: Sta-Lin group; fifth higher value: Und-Lin group; sixth higher value: Sta-Und group; seventh higher value: Sta-Lin group; eighth higher value: Und-Und group. The Kolmogorov-Smirnov test was performed to verify the normality of the groups.

We considered 5-day running per week in this study design, based on physical activities guidelines from the American College of Sports Medicine [64] that recommend healthy adults to practice cardiorespiratory training of moderate intensity between 30 and 60 min/day, ≥ 150 min/week in 5 days/week [45, 70, 71].

### Anthropometric Measurements

All participants were weighed by a mechanical scale (Filizola®, model 31, Brazil) with a capacity of 150 kg and precision of 50 g. ASIMED® Stadiometers (Spain), which presents a scale in millimeters used in order to obtain the participants’ height. The body mass index (BMI) was obtained by dividing weight (kg) per height (m^2^) (Quetelet’s equation). Body fat percentage (body fat %) was estimated from the measurement of skinfolds (triceps, subscapular, suprailiac, chest, abdominal, medium underarm, and thigh) using a Lange® compass, with an accuracy of 0.1 mm and predictive equations [72]. The equation developed by Jackson and Pollock [73] was used to calculate body density. Each skinfold was measured three times in a circuit, with the average of the three results considered as final index. The anthropometrical data collection before and after 8 weeks was conducted at the Exercise Physiology Laboratory (LaboFisE) of the Federal University of Rio de Janeiro, by a technician specialized in biometry and performed by the same evaluator with 7 years of experience in anthropometric analysis. The technical measurement error (ETM) that is, the variation in two different instants, was acceptable (ETM—relative: 2.1%).

### Cardiorespiratory Physical Fitness Test

The participants performed a cardiopulmonary exercise test with direct oxygen measurement (ergospirometry) on a treadmill (ECAFIX-EG700.2-Brazil). The flow measurement was obtained from the pneumotachograph (MEDGRAF) and the analysis of exhaled gas was performed using VO_2000_ (Inbrasport-Brazil). The following ventilatory variables were analyzed: minute ventilation (VE, l min^−1^—BTPS) and maximal oxygen uptake (V̇O_2max_, l min^−1^—STPD). Values of oxygen (FO_2_) and carbon dioxide (FCO_2_) were verified through calibration before and after the tests to correct possible deviations after exercise [74]. The calibration of the gas analyzer was performed by the device itself within 1 h prior to the start of the first evaluation, following the manufacturer’s recommendations (Pneumotachograph CPX, Medical Graphics Corp., St. Paul, MN). The gaseous samples were collected and measured every 10 s during the test.

All tests were performed under similar temperature conditions (22 °C and 24 °C), relative air humidity (between 40 and 60%), and barometric pressure of 760 mmHg. The day before the V̇O_2max_ analysis, participants were asked to avoid any strenuous exercise. Blood pressure (BP) was measured using the auscultatory method and heart rate (HR) was measured using electrodes (Micromed) placed at the manubrium and in the left and right iliac crest (derivation CM5), while participants were on the treadmill. The electrodes were connected to an electrocardiograph (Micromed) and HR values were visualized using the Elite software (Micromed Biotechnology, Brazil). The criteria used to find maximum oxygen consumption (V̇O_2max_) were a minimum of two of the following: (a) establishment of the oxygen uptake curve plateau relative to the load; (b) gas exchange ratio ≥ 1.1 [75]; (c) score ≥ 17 from 6 to 20 Borg’s scale [75]; and (d) maximum heart rate predicted for [208 − (0.7 × age)] [76]. The modified Bruce protocol was utilized for the cardiovascular testing [77]. This protocol includes 3-min stages with progressive loads determined by the increase in speed and incline, being 5 min of activity (1.60 mph) at 0% incline (warm up). The test was run from the first stage: 3 min, speed = 1.7 mph, slope = 0%; second stage: 3 min, speed = 2.5 mph, slope = 12%; third stage: 3 min, speed = 3.4 mph, slope = 14%; fourth stage: 3 min, speed = 4.2 mph, slope = 16%; fifth stage: 3 min, speed = 5.0 mph, slope = 18%; sixth stage: 3 min, speed = 5.5 mph, slope = 20%; seventh stage: 3 min, speed = 6.0 mph, slope = 22%.

### Blood Samples

Blood samples were obtained three times during the study: at baseline (pre-training), after 4 weeks, and after 8 weeks of training (post-training) in the beginning of the microcycle or 72 h after ending the 8th microcycle. Blood samples were collected at 8:00 am after 12 h of fasting. Three samples of 15 ml of blood were collected from the cephalic vein located in the antecubital fossa, and placed in tubes containing ethylenediaminetetraacetic acid (EDTA) without an anticoagulant for hematologic, biochemical, and hormonal analyses. Blood samples were maintained at − 2 °C to − 8 °C until the serum was separated by direct centrifugation at 3000 rpm for 15 min (Centra-8R IEC, MA). Serum samples were kept on ice and taken to freezer at − 20 °C until the time of analysis. The collection, storage, processing, and analysis of blood samples were performed by a specialized team consisting of a doctor and a nurse, both with experience in biochemical-hematological and hormonal tests.

### Blood Analysis

Serum levels of CK, LDH, U, aspartate aminotransferase (AST), and alanine aminotransferase (ALT) were performed by enzymatic methods using a Targa bt 3000® biochemical autoanalyzer. The biochemical reagents used were of the brand Wienner lab® (Wiener Lab, Rosario, Argentina). Concentrations of hemoglobin (Hb) and hematocrit (Ht) were measured by the spectrophotometric method using Cell-Dyn 3500 (System Operators Manual, Abbott Laboratories, Abbott Park, Illinois, USA). Mean corpuscular hemoglobin concentration (MCHC) was also calculated. Quantification of FT and cortisol (C) in the serum were done using kits (Elecsys 2010, Roche diagnostics, Germany) and analyzed by electrochemiluminescence and chemiluminescence, respectively, using an Hitachi Modular equipment (Hitachi, Tokyo, Japan). All analyses followed the protocol of their manufacturers and were performed in duplicates. Between and within coefficients of variation for all assays were less than 10% for all biochemical analyses.

### Statistical Analysis

The Kolmogorov-Smirnov test was used to check the normality of sample distribution and examined the appropriateness of using parametric tests. Analysis of variance (ANOVA) (mixed-design) was used to compare the differences between the “pre-training,” “after 4 weeks,” and “post-training” mean in the same group and between groups Und-Und, Und-Lin, Sta-Und, Sta-Lin versus control. Comparisons between the means of each variable studied, in the five groups were performed using ANOVA followed by the Tukey post-hoc test in order to identify the location of the differences in the dependent variables. Intra-class correlation coefficients (ICCs) were used to determine test-retest reliability. For the “pre-training” variables, effect size was calculated using Cohen’s d equation by subtracting the means of “post-training” and “pre-training” and dividing by the pooled “post-training” and “pre-training” standard deviations. The ranges adopted for small, medium, and large effects were 0.20, 0.50, and 0.80, respectively [78].

## Results

### Analyses of V̇O_2max_

**Fig. 3:**
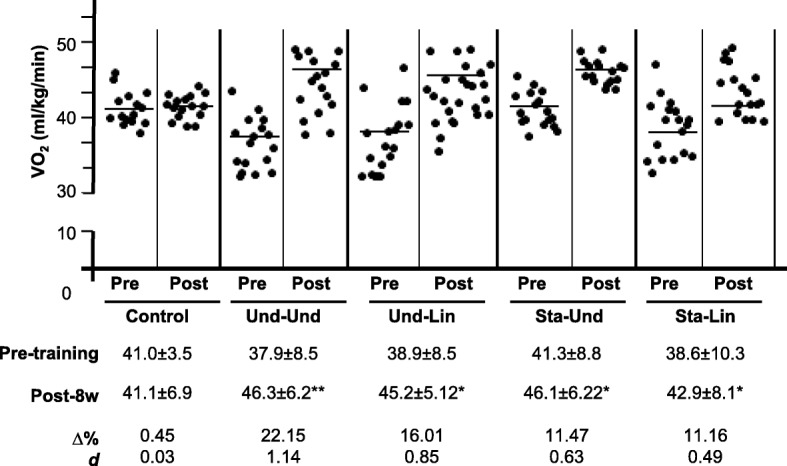
Effects of distinct aerobic trainings and taper-like periods on cardiorespiratory capacity of recreational athletes. Mean values of maximum oxygen consumption (V̇O_2max_) of healthy males before (pre) and after 8 weeks (post) of aerobic training. Groups: control (without training); undulatory-undulatory (Und-Und); undulatory-linear (Und-Lin); staggered-undulatory (Sta-Und); staggered-linear (Sta-Lin). Each black circle represents a participant in each group. Below graphs, the respective mean ± standard deviation values. *p ≤ 0.05. Δ, variation; *d*, effect size

In our study, we considered running as choice to evaluate cardiorespiratory capacity of the participants that were distributed in four different training groups and the control group based on Latin square design. In this context, the participants were distributed from the one with higher V̇O_2max_ to the one with lower V̇O_2max_ in the following sequence: Und-Und; Und-Lin; Sta-Und; Sta-Lin; and the control group. We did not find significant differences among the training groups and the control before (*p* = 0.95) and after 8 weeks of exercise (*p* = 0.71) (Fig. 3). In addition, no changes were observed in the cardiorespiratory fitness of the control group between pre-training and post-8 weeks of training (41.0 ± 3.5 versus 41.1 ± 6.9, respectively) (Fig. 3). However, evaluating the participants before and after training, we verified a trend to increase the mean values of the V̇O_2max_ in all training groups: Und-Und, 37.9 ± 8.5 versus 46.3 ± 6.2; Und-Lin, 38.9 ± 8.5 versus 45.2 ± 5.12; Sta-Und, 41.3 ± 8.8 versus 46.1 ± 6.22; Sta-Lin 38.6 ± 10.3 versus 42.9 ± 8.1, respectively comparing before and after 8 weeks of training (Fig. 3). No statistically significant differences were observed among the different training groups. The percentage changes and effect size calculations demonstrate that Und-Und group had the higher increase in V̇O_2max_ (Δ% = 22.15%, *d* = 1.14, *p* = 0.01) followed by Und-Lin (Δ% = 16.01, *d* = 0.85, *p* = 0.02), Sta-Und (Δ% = 11.47, *d* = 0.63, *p* = 0.02), and Sta-Lin groups (Δ% = 11.16, *d* = 0.49, *p* = 0.04) (Fig. 3).

### Circulating Levels of Hemoglobin and Hematocrit %

**Fig. 4:**
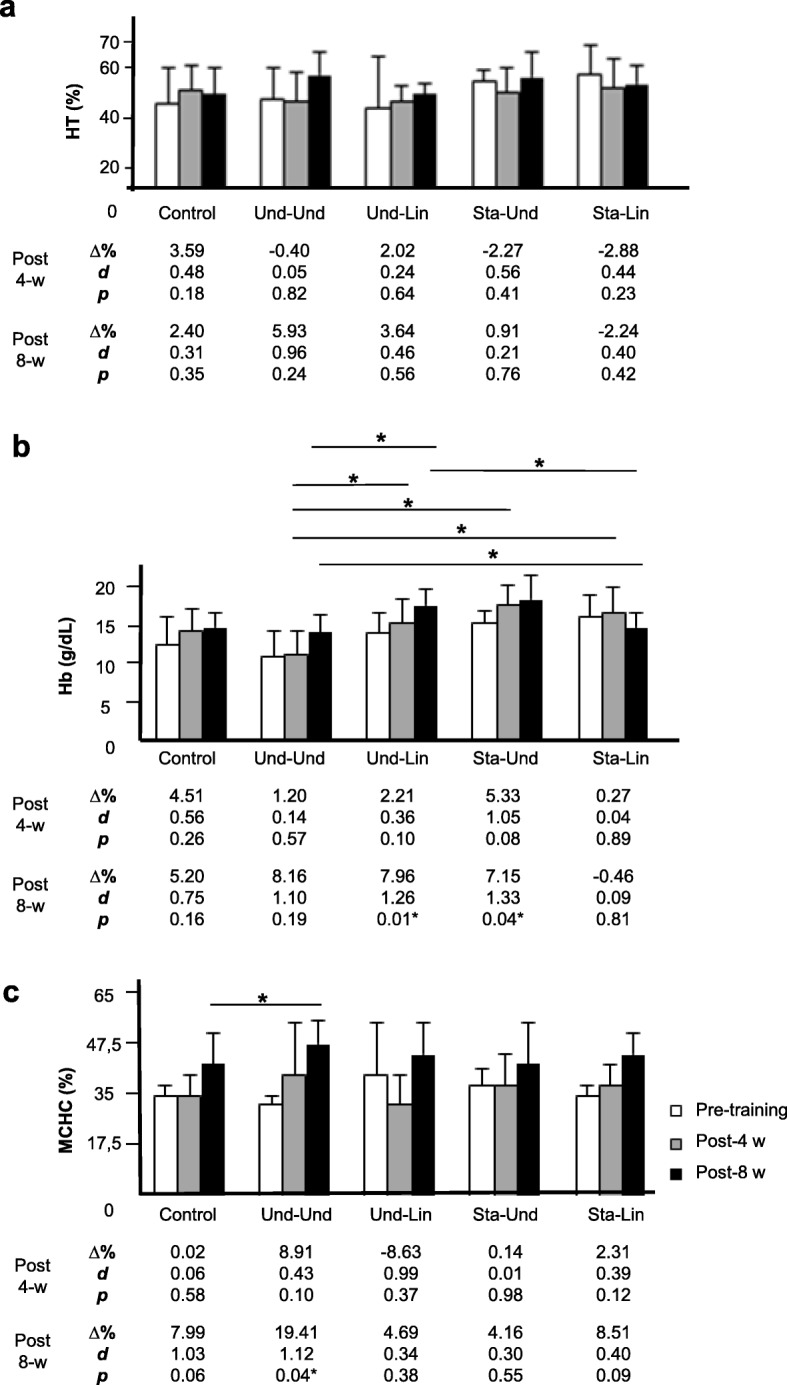
Effects of distinct aerobic trainings and taper-like periods on markers of O_2_ transport capacity to tissues in recreational athletes. Mean ± standard deviation values of **a** hematocrit %, **b** circulating levels of hemoglobin (HB), and **c** mean corpuscular hemoglobin concentration (MCHC) of recreational athletes before (white bar), post 4 (gray bar, 4-w) or 8 weeks (black bar, 8-w) of distinct aerobic trainings. Groups: control (without training), undulatory-undulatory (Und-Und), undulatory-linear (Und-Lin), staggered-undulatory (Sta-Und), and staggered-linear (Sta-Lin). Δ, variation; *d*, effect size; *indicates *p* ≤ 0.05. Statistical bars indicate comparisons between distinct groups

Our data showed no significant changes in Ht % among the distinct groups after 4 and 8 weeks of aerobic training (Fig. 4). However, we verified a positive trend to increase Ht % in the Und-Und group, in relation to the other models, 8 weeks post-training (Und-Und: 42.2 ± 3.1 versus 44.7 ± 2.2, Δ% = 5.93, *d* = 0.96, *p* = 0.24; Und-Lin: 41.2 ± 5.3 versus 42.7 ± 1.2, Δ% = 3.64, *d* = 0.46, *p* = 0.56; Sta-Und: 44.0 ± 1.2 versus 44.4 ± 2.61, Δ% = 0.91, *d* = 0.21, *p* = 0.76; Sta-Lin: 44.7 ± 3.0 versus 43.7 ± 2.0, Δ% = − 2,24, *d* = 0.40, *p* = 0.42; and control group 41.7 ± 3.8 versus 42.7 ± 2.7, Δ% = 2,40, *d* = 0.31, *p* = 0.35) (Fig. 4a). In relation to the plasma levels of Hb, no significant alteration was verified when comparing the distinct groups of load manipulation with the control after 4 weeks of training (Fig. 4b). However, after 8 weeks of training, Hb levels showed a trend to increase in proportion in the Und-Und group in comparison with the other training groups: Und-Und: 13.9 ± 1.2 versus 15.0 ± 0.8, Δ% = 8.16, *d* = 1.10, *p* = 0.19; Und-Lin: 15.0 ± 0.9 versus 16.2 ± 0.9, Δ% = 7.86, *d* = 1.26, *p* = 0.01; Sta-Und: 15.4 ± 0.6 versus 16.5 ± 1.1, Δ% = 7.15, *d* = 1.33, *p* = 0.04; Sta-Lin: 15.7 ± 1.0 versus 15.6 ± 0.7, Δ% = − 0.46, *d* = 0.09, *p* = 0.81; and control group 14.4 ± 1.3 versus 15.2 ± 0.7, Δ% = 5.20, *d* = 0.75, *p* = 0.16. After 8 weeks of training, Sta-Und model showed a lower gain in Ht and a trend to increase Hb levels while Sta-Lin group showed a trend to decrease Ht % and Hb levels. On the other hand, Und-Und group showed a trend to increase Ht % and Hb levels. Evaluating the MCHC, only Und-Und showed a significant increase in comparison with the control group, 8 weeks post aerobic training (Fig. 4c): Und-Und: 34.1 ± 1.3 versus 40.2 ± 2.3, Δ% = 19.41, *d* = 1.12, *p* = 0.04; Und-Lin: 37.3 ± 5.0 versus 39.2 ± 3.1, Δ% = 4.69, *d* = 0.34, *p* = 0.38; Sta-Und: 36.2 ± 1.5 versus 38.4 ± 4.1, Δ% = 4.16, *d* = 0.30, *p* = 0.55; Sta-Lin: 35.0 ± 0.9 versus 39.0 ± 1.8, Δ% = 8.51, *d* = 0.40, *p* = 0.09; and control group 35.2 ± 1.4 versus 38.0 ± 2.8, Δ% = 7.99, *d* = 1.03, *p* = 0.06. We did not find significant difference in intergroups. These data suggest that the Und-Und load manipulation model was the one that most contributed for oxygen supply in recreational athletes.

### Body Mass Index and Body Fat %

As shown in Table 1, our analyses indicate that the BMI mean in all groups in the pre-training phase, before the aerobic training, varied from 25.1 to 26.0 kg/m^2^ being not statistically different among groups (*p* = 0.99). Moreover, we verified that after 8 weeks of training, the BMI values of Und-Und, Und-Lin, Sta-Und, and Sta-Lin groups and the control varied from 24.8 to 26.2 kg/m^2^ (*p* = 0.98). We found the following BMI mean values for each group: Und-Und, 25.8 ± 4.6 versus 24.9 ± 4.4, Δ% = − 8.70, *d* = 0.05; Und-Lin, 25.8 ± 5.2 versus 25.7 ± 5.3, Δ% = − 0.32, *d* = 0.02; Sta-Und, 25.1 ± 3.0 versus 24.8 ± 3.0, Δ% = − 0.87, *d* = 0.10; Sta-Lin 25.9 ± 4.6 versus 25.8 ± 4.4, Δ% = − 0.12, *d* = 0.02; and control group: 26.0 ± 0.4 versus 26.2 ± 0.7, Δ% = 0.66, *d* = 0.35, comparing before and after 8 weeks of training (Table 1).

**Table 1:** Mean values for age, BMI, and body fat % of recreational athletes submitted to short-term aerobic training programs

Variables.	Groups					*p* value
	Control (*n* = 16)	Und-Und (*n* = 18)	Und-Lin (*n* = 19)	Sta-Und (*n* = 17)	Sta-Lin (*n* = 18)	
Pre-training						
Age	29.0 ± 0.7	27.7 ± 9.3	26.3 ± 6.5	26.6 ± 7.2	24.3 ± 4.0	0.86
Range (years)	28–30	20–35	21–35	20–35	22–32	
CV (%)	2.41	35.02	24.71	27.07	16.46	
BMI	26.0 ± 0.4	25.8 ± 4.6	25.8 ± 5.2	25.1 ± 3.0	25.9 ± 4.6	0.99
Range (kg/m^2^)	21.33–30.47	20.76–29.92	20.87–28.38	21.21–27.86	19.45–29.04	
CV (%)	1.54	17.83	20.15	11.95	17.76	
Body fat	15.3 ± 1.9	14.5 ± 5.8	11.1 ± 5.9	10.4 ± 4.3	13.1 ± 3.8	0.58
Range (%)	11.86–22.15	12.37–24.77	9.18–24.83	10,15–23.34	12.88–24.31	
CV (%)	12.42	40	53.15	41.35	29.01	
Post-training (8 weeks)						
BMI	26.2 ± 0.7	24.9 ± 4.4 *	25.7 ± 5.3*	24.8 ± 3.0	25.8 ± 4.4 *	0.98
Range (kg/m^2^)	20.81–28,53	19.65–27.90	20.30–26.36	20.59–28.95	20.19–27.18	
CV (%)	2.67	17.67	20.62	12.10	17.05	
Δ (%)	0.66	− 8.70	− 0.32	− 0.87	− 0.12	
*d*	0.35	0.05	0.02	0.10	0.02	
body fat	16.5 ± 2.9	13.2 ± 6.3*	9.2 ± 5.0	8.6 ± 2.9	10.9 ± 6.3*	0.16
Range (%)	14.8–20.6	9.45–17.30	8.31–15.73	8.14–12.32	8.89–16.12	
CV (%)	17.57	47.73	54.35	33.72	59.80	
Δ (%)	7.85	− 8.78	− 17.58	− 17.08	− 16.45	
*d*	0.47	0.21	0.36	0.49	0.33	
*p*	0.17	0.04	0.06	0.09	0.03	

*BMI* body mass index, Δ variation, *d* effect size. Values are indicated as mean ± standard deviation; * significant difference (*p* ≤ 0.05)

Analyzing the body fat % of the participants inside each group, before and after 8 weeks of aerobic training, we observed a significant decrease in percent variation in the Und-Und (before: 14.5 ± 5.8; post-training: 13.2 ± 6.3; Δ% = − 8.78, *d* = 0.21; *p* = 0.04) and in the Sta-Lin group (before: 13.1 ± 3.8; post-training: 10.9 ± 6.3; Δ% = − 16.45, *d* = 0.33, *p* = 0.03) while a trend to decrease the percent variation was verified in the Und-Lin (before: 11.1 ± 5.9; post-training: 9.2 ± 5.0; Δ% = − 17.58, *d* = 0.36, *p* = 0.06) and Sta-Und (before: 10.4 ± 4.3; post-training: 8.6 ± 2.9; Δ% = − 17.08, *d* = 0.49, *p* = 0.09) groups (Table 1). No significant changes were observed in the control group. The group with staggered manipulation in the first mesocycle and linear load in the second mesocycle (Sta-Lin) did not change the BMI mean, although promoted a significant decrease in the body fat % of the participants. The groups with undulatory load in the first mesocycle and linear load in the second mesocycle (Und-Lin) or with staggered manipulation in the first mesocycle and undulatory (Sta-Und) load in the second mesocycle did not significantly change the BMI and the body fat % of the participants (Table 1).

### Alterations in the Circulating Levels of Muscle Injury Markers

**Fig. 5:**
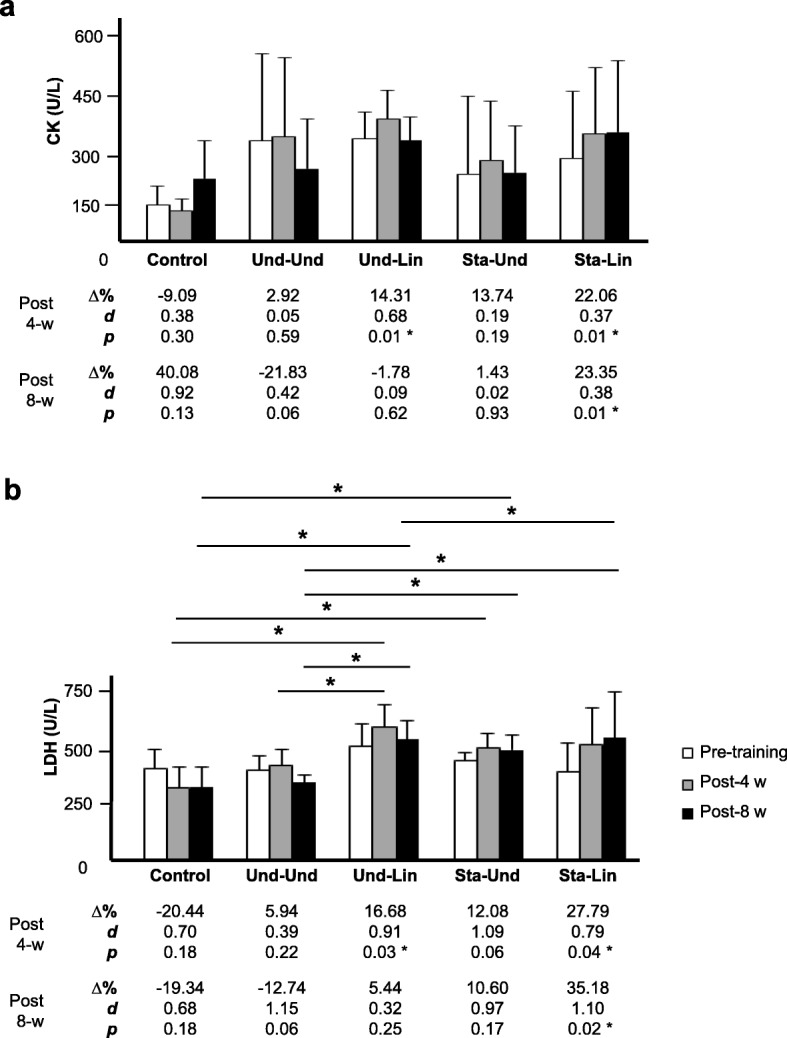
Effects of distinct aerobic trainings and taper-like periods on muscle stress markers in recreational athletes. Mean ± standard deviation values of **a** creatine kinase (CK) and **b** lactate dehydrogenase (LDH) circulating levels in recreational athletes, before (white bar), post 4 (gray bar, 4-w) or post 8 weeks (black bar, 8-w) of distinct aerobic trainings, as indicated in the legend. Groups: control (without training), undulatory-undulatory (Und-Und), undulatory-linear (Und-Lin), staggered-undulatory (Sta-Und), and staggered-linear (Sta-Lin). Δ, variation; *d*, effect size; * indicates *p* ≤ 0.05. Statistical bars indicate comparisons between different groups

When we evaluated the circulating levels of CK in the four groups of participants after 4 weeks of aerobic training, Und-Und (359.7 ± 239.4 versus 370.2 ± 218.3, Δ% = 2.92%, *d* = 0.05, *p* = 0.59) did not significantly change (Fig. 5a). In the control, the circulating levels of CK did not change (151.5 ± 53.0 versus 135.0 ± 32.9, Δ% = − 9.09% *d* = 0.38, *p* = 0.30) (Fig. 5a). Sta-Und (266.4 ± 216.0 versus 303.3 ± 165.7, Δ% = 13.74%, *d* = 0.19, *p* = 0.19) showed a non-significant increase while the Und-Lin (365.7 ± 72.4 versus 418.0 ± 81.6, Δ% = 14.31%, *d* = 0.68, *p* = 0.01) and Sta-Lin (310.1 ± 185.4 versus 378.6 ± 183.6, Δ% = 22.06%, *d* = 0.37, p = 0.01) groups increased significantly the circulating levels of CK (Fig. 5a). From the week 1 to 8 post-training, the Und-Und group did not significantly change the CK levels (359.7 ± 239.4 versus 281.2 ± 136.6, Δ% = − 21.83%, *d* = 0.42, *p* = 0.06). No change was verified in the mean levels of CK in the Und-Lin (365.7 ± 72.4 versus 359.2 ± 65.2, Δ% = − 1.78%, *d* = 0.09, *p* = 0.62) and Sta-Und (266.4 ± 216.0 versus 270.2 ± 129.1, Δ% = 1.43%, *d* = 0.02, *p* = 0.93) groups. However, Sta-Lin group showed a significant increase in the mean values of CK (310.1 ± 185.4 versus 382.6 ± 197.7, Δ% = 23.35%, *d* = 0.38, *p* = 0.01). In the control, we did not verify significant change in the circulating levels of CK (151.5 ± 53.0 versus 254.2 ± 105.6, Δ% = 40.08%, *d* = 0.92, *p* = 0.13) (Fig. 5a).

In relation to mean values of LDH enzyme, we observed that in all four groups there was an increase from the week 1 to 4 after aerobic training (Fig. 5b). In the control, we verified a trend to decrease circulating LDH levels (411.0 ± 150.7 versus 327.0 ± 89.8, Δ% = − 20.44, *d* = 0.70, *p* = 0.18), which could be explained by the lack of training during the 8-week period. While the Und-Und showed a lower trend in relation to Sta-Und to increase the levels of LDH (Und-Und: 404.2 ± 60.0 versus 428.2 ± 63.5, Δ% = 5.94, *d* = 0.39, *p* = 0.22), (Sta-Und: 445.2 ± 34.9 versus 499.0 ± 63.7, Δ% = 12.08, *d* = 1.09, p = 0.06), a significant increase was verified in the Und-Lin and, mainly, in the Sta-Lin group (Und-Lin: 508.5 ± 92.0 versus 593.3 ± 94.9, Δ% = 16.68, *d* = 0.91, *p* = 0.01) (Sta-Lin: 402.0 ± 121.2 versus 513.7 ± 159.9, Δ% = 27.79, *d* = 0.79, *p* = 0.04) (Fig. 5b). Physiologically, it can be associated with direct muscle injury in individuals submitted to the model without periodization. From the week 1 to 8 post-training, the LDH levels showed a trend to be decreased in the Und-Und group (404.2 ± 60.0 versus 352.7 ± 29.2, Δ% = − 12.74%, *d* = 1.15, *p* = 0.06) or increased in the Und-Lin (508.5 ± 92.0 versus 536.2 ± 81.6, Δ% = 5.44, *d* = 0.32, *p* = 0.25) and Sta-Und groups (445.2 ± 34.9 versus 492.4 ± 62.6, Δ% = 10.60%, *d* = 0.97, *p* = 0.17). In the Sta-Lin group, we verified a significant increase in the circulating levels of LDH (402.0 ± 121.2 versus 543.4 ± 135.4, Δ% = 35.18, *d* = 1.10, *p* = 0.02) (Fig. 5b).

**Fig. 6:**
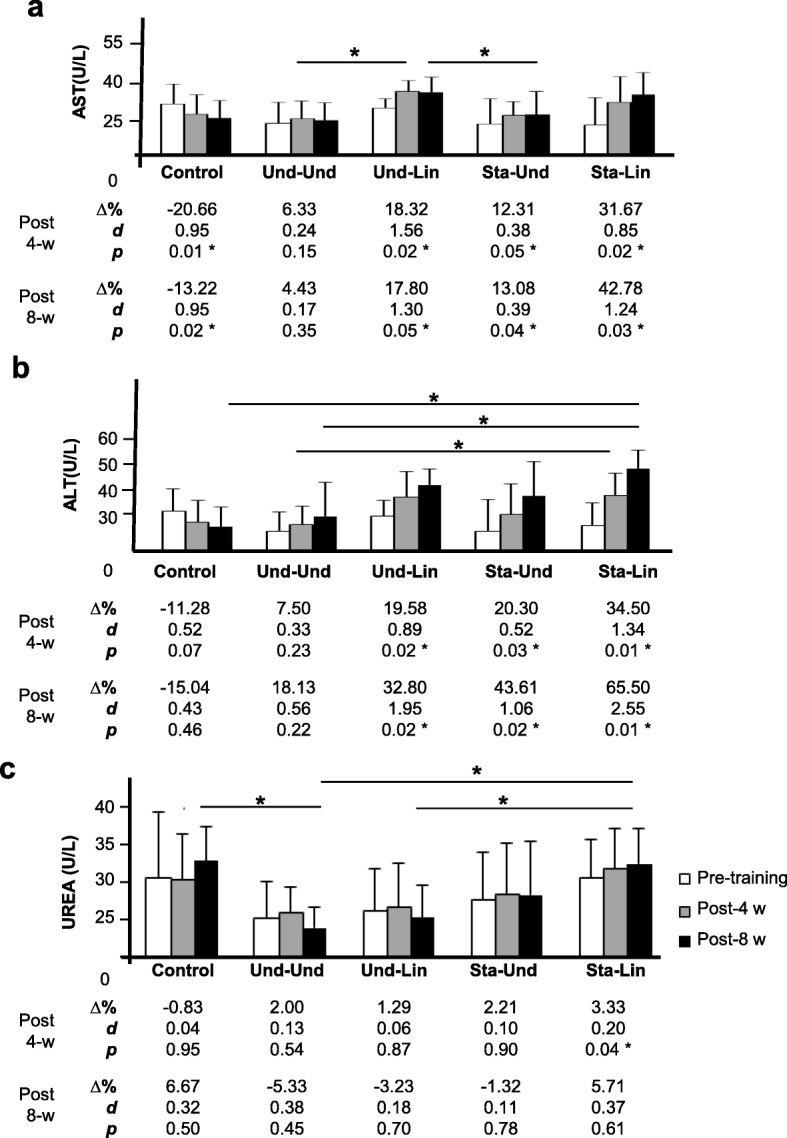
Alterations in the circulating levels of liver enzymes and urea in recreational athletes submitted to distinct aerobic trainings with taper-like periods. Mean ± Standard deviation values of **a** aspartate aminotransferase (AST), **b** alanine aminotransferase (ALT), and **c** urea (U) circulating levels in recreational athletes, before (white bar), post 4 (gray bar, 4-w) or post 8 weeks (black bar, 8-w) of distinct aerobic trainings, as indicated in the legend. Groups: control (without training), undulatory-undulatory (Und-Und), undulatory-linear (Und-Lin), staggered-undulatory (Sta-Und), and staggered-linear (Sta-Lin). Δ, variation; *d*, effect size; * indicates *p* ≤ 0.05. Statistical bars indicate comparisons between different groups

After 4 weeks of training, Und-Lin (31.8 ± 3.4 versus 37.7 ± 4.03, Δ% = 18.32, *d* = 1.56, *p* = 0.03), Sta-Und (26.00 ± 9.2 versus 29.2 ± 4.8, Δ% = 12.31, *d* = 0.38, *p* = 0.05), and Sta-Lin (25.7 ± 9.9 versus 33.9 ± 9.3, Δ% = 31.67, **d** = 0.85, *p* = 0.02) groups showed a significant increase in the levels of AST while Und-Und (26.3 ± 7.6 versus 28.0 ± 6.2, Δ% = 6.33, *d* = 0.24, *p* = 0.15) showed only a low trend to increase circulating levels of AST (Fig. 6a). Comparing week 1 to 8, the levels of AST were also increased mainly in the Und-Lin (31.8 ± 3.4 versus 37.5 ± 5.3, Δ% = 17.80, *d* = 1.30, *p* = 0.05), Sta-Und (26.00 ± 9.2 versus 29.4 ± 8.4, Δ% = 13.08, *d* = 0.39, *p* = 0.04), and Sta-Lin groups (25.7 ± 9.9 versus 36.7 ± 7.8, Δ% = 42.78, *d* = 1.24, *p* = 0.02) (Fig. 6a), while Und-Und (26.30 ± 7.60 versus 27.50 ± 6.10, Δ% = 4.43, *d* = 0.17, *p* = 0.35) showed a low increase, comparatively. In addition, a non-significant low increase was verified in the Und-Und group (26.7 ± 6.1 versus 28.7 ± 6.0, Δ% = 7.50, *d* = 0.33, *p* = 0.23) in the circulating levels of ALT after 4 weeks of training (Fig. 6b). In all other groups, there was a significant increase in the levels of ALT: Und-Lin (31.5 ± 5.2 versus 37.7 ± 5.38.7, Δ% = 19.58, *d* = 0.52, *p* = 0.04), Sta-Und (26.60 ± 10.5 versus 32.00 ± 10.20, Δ% = 20.32, *d* = 0.89, *p* = 0.03), and Sta-Lin (28.6 ± 7.6 versus 38.4 ± 7.4, Δ% = 34.50, *d* = 1.34, *p* = 0.01) groups (Fig. 6b). After 8 weeks of aerobic training, excepting the Und-Und group (26.7 ± 6.1 versus 31.5 ± 11.1, Δ% = 18.13, *d* = 0.56, *p* = 0.22), the circulating levels of ALT significantly increased in all groups: Und-Lin (31.5 ± 5.2 versus 41.8 ± 5.30, Δ% = 32.80, *d* = 1.95, *p* = 0.03), Sta-Und (26.6 ± 10.5 versus 38.2 ± 11.30, Δ% = 43.61, *d* = 1.08, *p* = 0.02), and Sta-Lin (28.6 ± 7.3 versus 47.3 ± 7.40, Δ% = 65.50, *d* = 2.55, *p* = 0.01) (Fig. 6b).

After 4 weeks of training, we did not find significant changes in urea (U) levels in the Und-Und (25.0 ± 4.40 versus 25.5 ± 3.20, Δ% = 2.00, *d* = 0.13, *p* = 0.54), Und-Lin (25.80 ± 5.20 versus 26.2 ± 5.50, Δ% = 1.29, *d* = 0.06, *p* = 0.87), and Sta-Und (27.2 ± 6.0 versus 27.8 ± 6.50, Δ% = 2.21, *d* = 0.10, *p* = 0.90) groups, although we found a significant increase in the U levels in individuals of the Sta-Lin group (30.0 ± 4.8 versus 31.0 ± 5.00, Δ% = 3.33, *d* = 0.20, *p* = 0.04) (Fig. 6c). From week 1 to 8, no changes were detected in the circulating levels of U in all groups evaluated: Und-Und (25.0 ± 4.4 versus 23.7 ± 2.50, Δ% = − 5.33, *d* = 0.38, *p* = 0.45), Und-Lin (25.8 ± 5.2 versus 25.00 ± 4.00, Δ% = − 3.23, *d* = 0.18, *p* = 0.70), Sta-Und (27.2 ± 6.00 versus 27.8 ± 6.60, Δ% = 2.21, *d* = 0.10, p = 0.90), or Sta-Lin (30.0 ± 4.80 versus 31.70 ± 4.40, Δ% = 5.71, *d* = 0.37, *p* = 0.61) (Fig. 6c).

These data suggest that the Und-Und group had a better efficacy in the recovery of the aerobic training while the Sta-Lin group did not recover well as indicated by the accumulation of serum CK, LDH, AST, ALT enzymes, and U levels.

### Circulating Levels of Cortisol, Testosterone, and FT/C Ratio

**Fig. 7:**
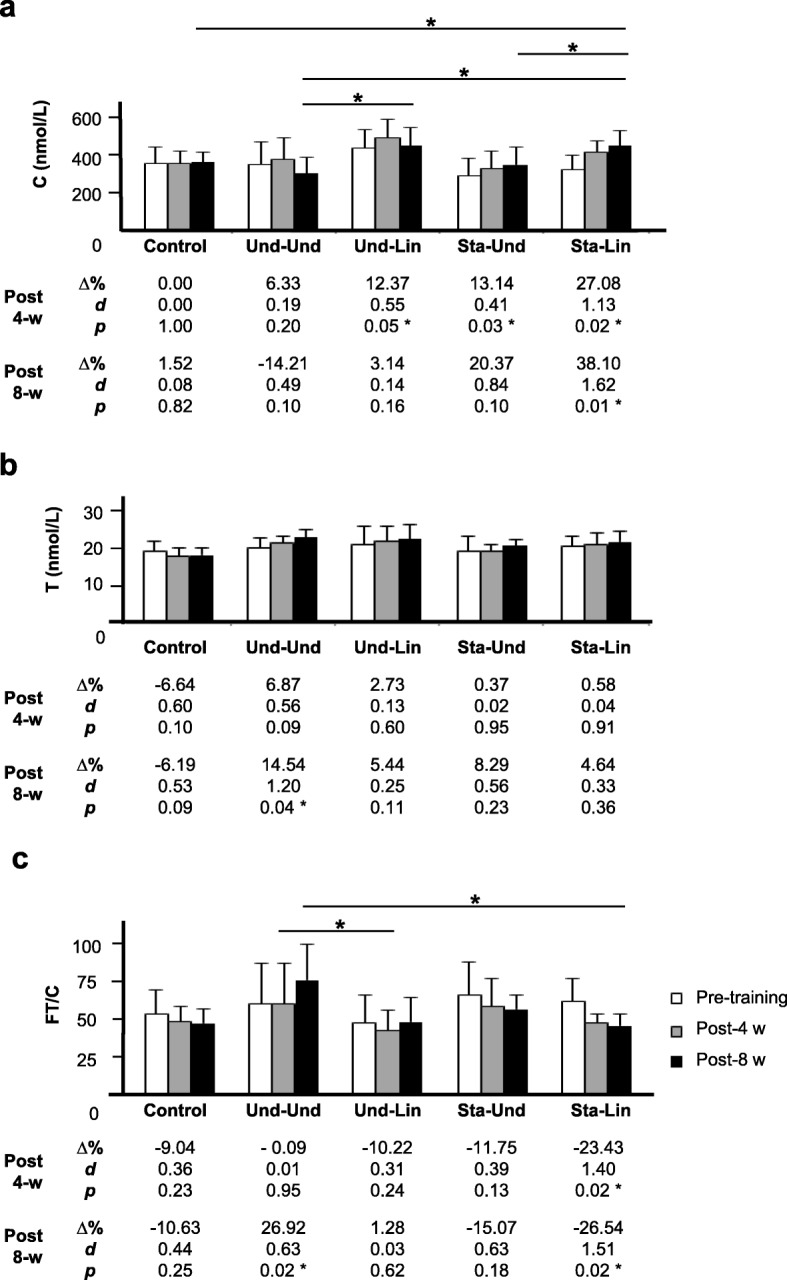
Effects of distinct aerobic trainings and taper-like periods on markers for muscle catabolism and anabolism in recreational athletes. Mean ± standard deviation values of **a** cortisol (C) and **b** testosterone (T) circulating levels and of **c** free testosterone/cortisol ratio (FT/C) in recreational athletes, before (white bar), post 4 (gray bar, 4-w) or post 8 weeks (black bar, 8-w) of distinct aerobic trainings, as indicated in the legend. Groups: control (without training), undulatory-undulatory (Und-Und), undulatory-linear (Und-Lin), staggered-undulatory (Sta-Und), and staggered-linear (Sta-Lin). Δ, variation; *d*, effect size; * indicates *p* ≤ 0.05. Statistical bars indicate comparisons between different groups

We verified only a trend to increase C circulating levels after 4 weeks of aerobic training in the Und-Und group (359.20 ± 119.20 versus 381.90 ± 119.00, Δ% = 6.33, *d* = 0.19, *p* = 0.20) (Fig. 7a). On the other hand, a significant increase in the C levels was observed in recreational athletes after 4 weeks of aerobic exercise in all other groups: Und-Lin (443.30 ± 101.30 versus 498.10 ± 97.40, Δ% = 12.37, *d* = 0.55, *p* = 0.05), Sta-Und (295.20 ± 95.20 versus 334.00 ± 92.60, Δ% = 13.14, *d* = 0.41, *p* = 0.03), and Sta-Lin (331.10 ± 76.40 versus 420.70 ± 59.60, Δ% = 27.08, *d* = 1.13, *p* = 0.01) (Fig. 7a). When comparing from week 1 to 8, we found a trend to decrease the levels of C in the Und-Und group (359.20 ± 119.20 versus 308.10 ± 89.80, Δ% = − 14.21, *d* = 0.49, *p* = 0.10) and a non-significant increase in the circulating levels of this hormone in the Und-Lin (443.30 ± 101.30 versus 457.20 ± 94.90, Δ% = 3.14, *d* = 0.14, *p* = 0.16) and Sta-Und (295.2 ± 95.30 versus 355.30 ± 48.00, Δ% = 20.37, *d* = 0.84, *p* = 0.10) groups. A significant increase in the circulating levels of C was verified in the Sta-Lin group (331.10 ± 76.40 versus 457.20 ± 80.60, Δ% = 38.10, *d* = 1.62, *p* = 0.01) (Fig. 7a).

Evaluating the circulating levels of FT, we verified that after 4 weeks, no significant change was observed in all groups: Und-Und (20.30 ± 2.90 versus 21.70 ± 2.10, Δ% = 6.87, *d* = 0.56, *p* = 0.09), Und-Lin (21.60 ± 5.00 versus 22.20 ± 4.20, Δ% = 2.73, *d* = 0.13, *p* = 0.60), Sta-Und (19.50 ± 4.00 versus 19.60 ± 1.80, Δ% = 0.37, *d* = 0.02, *p* = 0.95), and Sta-Lin (21.10 ± 2.70 versus 21.20 ± 3.40, Δ% = 0.58, *d* = 0.04, *p* = 0.91) (Fig. 7b). However, from week 1 to 8, we found a significant increase in the circulating FT levels only in the Und-Und group (20.30 ± 2.90 versus 21.70 ± 2.10, Δ% = 14.54, *d* = 1.20, *p* = 0.04) while a trend to increase the levels of this anabolic hormone was verified in the Und-Lin (21.60 ± 5.00 versus 22.80 ± 4.30, Δ% = 5.44, *d* = 0.25, *p* = 0.11), Sta-Und (19.50 ± 4.00 versus 21.10 ± 1.80, Δ% = 8.29, *d* = 0.56, *p* = 0.23), and Sta-Lin (21.10 ± 2.70 versus 22.10 ± 3.10, Δ% = 4.64, *d* = 0.33, *p* = 0.36) groups (Fig. 7b).

After 4 weeks, no change in the FT/C ratio was verified in the Und-Und group (64.40 ± 29.00 versus 64.40 ± 29.20, Δ% = − 0.02, *d* = 0.01, *p* = 0.98) (Fig. 7c). On the other hand, we observed a trend to decrease the FT/C ratio in Und-Lin (51.30 ± 20.20 versus 46.00 ± 13.70, Δ% = − 10.22, *d* = 0.31, *p* = 0.24) and Sta-Und (71.40 ± 23.00 versus 63.80 ± 20.00, Δ% = − 11.75, *d* = 0.39, *p* = 0.13) group. A significant decrease in the FT/C ratio was found in the Sta-Lin group (66.50 ± 16.80 versus 50.90 ± 6.10, Δ% = − 23.43, *d* = 1.40, *p* = 0.02) (Fig. 7c). After 8 weeks, only Und-Und group showed a significant increase in the FT/C ratio (64.40 ± 29.00 versus 81.80 ± 26.10, Δ% = 26.92, *d* = 0.63, *p* = 0.02). No change in the FT/C ratio was observed in the Und-Lin group (51.30 ± 20.20 versus 51.90 ± 17.60, Δ% = 1.28, *d* = 0.03, *p* = 0.62). However, a trend to decrease this ratio was found in the Sta-Und group (71.40 ± 23.00 versus 60.60 ± 10.80, Δ% = − 15.07, *d* = 0.63, *p* = 0.18). The FT/C ratio significantly decreased in the Sta-Lin group (66.50 ± 16.80 versus 48.90 ± 8.10, Δ% = − 26.54, *d* = 1.51, *p* = 0.02) (Fig. 7c). These results indicate that Sta-Lin model increased catabolism and was physiological deleterious, promoting more muscle injury and inefficient recovery as well as confirmed Und-Und as the best model for muscle recovery in recreational athletes submitted to distinct short-term aerobic training (Table 2).

**Table 2:** Summary of alterations in V̇O_2max_, hematological, biochemical, and endocrine markers after 8 weeks of aerobic training in recreational athletes submitted to distinct periodization models

	V̇O_2max_ (ml/kg/min) Mean	Hb (g/dl) ∆%	Ht (%) ∆%	MCHC (%) ∆%	CK (U/L) ∆%	LDH (U/L) ∆%	AST (U/L) ∆%	ALT (U/L) ∆%	Urea (U/L) ∆%	C (nmol/L) ∆%	T (nmol/L) ∆%	FT/C ratio
Periodization												
Und-Und	↑ *	↑	↑	↑*	↓	↓	↑	↑	↓	↓	↑*	↑*
Und-Lin	↑ *	↑*	↑	↑	=	↑	↑*	↑*	↓	↑	↑	=
Sta-Und	↑ *	↑*	=	↑	=	↑	↑*	↑*	↑	↑	↑	↓
Sta-Lin	↑ *	=	↓	↑	↑*	↑*	↑*	↑*	↑	↑*	↑	↓*

* Indicates statistical difference (*p* ≤ 0.05) in mean values, **∆**% or FT/C ratio between the pre-training and post-training period; ↑ means trend to increase; ↓ means trend to decrease; = means similar values between the pre-training and post-training period

## Discussion

We investigated the aerobic fitness of healthy males after aerobic exercise using a novel model of analysis, where tapering-like period was applied in the final week of distinct aerobic training models, including undulatory and non-undulatory load manipulations. We evaluated V̇O_2max_ and the circulating levels of hematological, biochemical, and hormonal markers before, during, and after aerobic training of recreational athletes. Our data indicate that after 8 weeks of training, (a) the greater cardiorespiratory fitness among all training groups was associated with the group that followed undulatory load manipulation with both daily and weekly loads (the und-und group) and reduced load in the final week, (b) the lower muscle injury and best recovery was also verified in the Und-Und group, and (c) staggered load manipulation, without load reduction in the final week of training, seems to promote inadequate recovery, as mainly observed in the Sta-Lin group.

In our study, we were limited to use V̇O_2max_ as parameter to prescribe exercise intensity to improve cardiorespiratory fitness [66–69]. Different groups indicated a low correlation between Fc_max_ and % of V̇O_2max_. In this regard, Buchheit et al. verified that heart rate (HR) at rest during and after exercise tests served as parameters to verify the aerobic performance in healthy runners [79]. However, a large percentage variation was observed in the % of V̇O_2max_ (2 to 19%) of health men, after an individual endurance training program [80]. On the other hand, Katch et al. analyzed the % of V̇O_2max_, heart rate, and metabolic acidosis in the intensity of 60%, 70%, and 80% in 31 individuals submitted to a progressive cycle ergometer test (V̇O_2max_ range was between 37.8 and 68.8). These authors verified that 60%, 70%, and 80% of the maximum heart rate corresponded to 36%, 48%, and 62% of V̇O_2max_, respectively, and concluded that metabolic acidosis is not constant among the participants exercising at the same relative percentage of HRmax, thus indicating a low prediction between % of V̇O_2max_ and HR [81]. In this regard, it is worth noting that Pollock previously found similar values [82]. Additionally, Wolpern et al. after observing healthy but sedentary men and women (*n* = 36) in a treadmill test concluded that the anaerobic threshold was the most efficient parameter to analyze exercise intensity when compared to % of HR reserve [56]. Although some authors indicate a low correlation between Fcmax and % of V̇O_2max_, Garber indicated that the intensity of V̇O_2max_ in percentages (64% to 91%) corresponds to 76% to 96% of Fcmax %, respectively, among individuals of 20 to 39 years of age [45]. Furthermore, the American College of Sport Medicine [64] recommend that the appropriate intensity to increase cardiorespiratory fitness for healthy adults should be 60–90% of Fcmax, which correspond to 50–85% of maximum oxygen consumption (V̇O_2max_). On the other hand, these variables are not related to the individual metabolic responses of blood lactate, and can vary individually, as a form of adaptation, in each exercise session [83].

Despite all training groups showing significant improvement in V̇O_2max_, after 8 weeks of training, the gain (22.15%) and the largest effect size (*d* = 1.14) in V̇O_2max_ were more evidenced in the Und-und group. Based on Δ% comparison, Und-Lin group presented greater gain (16.01%) than Sta-und group (11.47%) while Sta-Lin group presented the lowest improvement in V̇O_2max_ (11.16%). These findings are in agreement with our previous work [57] showing a significant increase in the aerobic fitness of these same training groups of recreational athletes after 8 weeks of running, where the Und-und group showed the better distance performance [57]. We could explain the greatest improvement in V̇O_2max_ in the Und-Und and Und-Lin groups by the reduction in the weekly volume of training, as our group previously suggested, considering the intervention with a microcycle for best recovery, avoiding detraining [57]. In agreement with our data, an increase of 7.92% of V̇O_2max_ was observed in recreational exercise practitioners after a week of taper period [51]. In another study with recreational runners, an increase of 5.05% in V̇O_2max_ was observed after 13 weeks of training followed by 3 weeks of taper [52]. Other researchers verified a V̇O_2max_ gain of 3.07% in athletes following long-distance running and 7 days of tapering, with 85% of volume reduction without decreasing weekly training sessions [33]. Moreover, a review showed that of 13 articles analyzed, ten demonstrated increased V̇O_2max_ with training tapering varying from 7 until 28 days; two showed no effect and one showed a trend to reduce V̇O_2max_ in athletes, including swimmers, cyclist, triathletes, and runners [49]. These data indicate that tapering ameliorates aerobic fitness in recreational runners as that observed in athletes.

In our study, we verified that recreational athletes submitted to Und-Und and Und-Lin taper-like periods present high increase in V̇O_2max_ gain and trend to increase Ht% and Hb circulating levels after 8 weeks of training. Schumacher and colleagues [84] showed an association between the regular practice of exercise with elevated circulating levels of Hb and Ht % with improvement in O_2_ transport and V̇O_2max_ in athletes, a condition that can be altered according to the exercise intensity. In our study, it is possible to consider that a reduced volume of training in the Und-Und and Und-Lin taper-like periods could decrease hemolysis [26, 33, 85] induced by aerobic training for 8 weeks with the final week of tapering-like period. Yamamoto and colleagues [3] reported increased mean concentrations of Hb and Ht % in young swimmers after the tapering off period of 1 week. On the other hand, another group showed that male swimmers presented a trend to decrease Ht %, Hb levels and a significant reduction of MCHC after 4 weeks of taper [86]. However, Mujika and collaborators [85] verified an increase of MCHC in athletes submitted to high load of training with a decrease in the taper period of 4 weeks. The authors also observed a trend to decrease Hb levels in the final taper period. These studies suggest that the taper duration, as well as the time of previous training, might influence negatively on O_2_ consumption in both athletes and recreational athletes.

We verified a trend to reduce the levels of Hb and Ht % in the Sta-Lin group. Possibly, an increased volemia [87] could occur in the Sta-Lin group due to an inefficient recovery. In this regard, other groups have shown a trend to decrease Ht% and the Hb circulating levels in competitive swimmers submitted to intense training [3, 88]. Moreover, it has been described that decreased Ht% can occur in athletes using elevated loads of endurance training in association with plasma volume expansion [89]. For instance, it could lead to increase in the levels of aldosterone and activation of plasmatic proteins [90] and to a reduction in Fe^++^ levels, as observed in long distance runners [91].

In relation to body fat % after aerobic training, we observed a significant decrease in all groups. The BMI showed a trend to decrease more in the Und-Und group than in the Und-Lin, Sta-Und, and Sta-Lin groups. Other studies also verified a trend to decrease body fat % and BMI in recreational athletes following aerobic training periodization with moderate intensity [57, 92, 93]. In addition, it was verified an inverse correlation between BMI and body fat % with V̇O_2max_ levels while the free fat mass was positively correlated with the V̇O_2max_ [94]. In this scenarium, regular activity might contribute to decrease body fat % leading to an increase in free fat mass and V̇O_2max_ levels [95].

In our study, we measured the circulating levels of CK and LDH in recreational athletes submitted to 8 weeks of training. We observed that the groups that had taper-like periods in their training showed a trend to decrease the circulating levels of CK while Sta-Lin group increased the circulating levels of this enzyme. In this context, it is known that running is considered predominantly an eccentric action, which means that the load is above muscle capacity to sustain it promoting more muscle microlesions in comparison with concentric and isometric actions [96, 97]. These muscle microlesions can lead to “hemolysis by impact” [98]. Physiologically, these lesions could be explained by the higher passive tension of the connective tissue on the muscle fibers [99, 100]. In addition, eccentric actions recrute less motor units, i.e., alpha-neurons, than concentric actions but induces elevated mechanic stress in the muscle fibers thus leading to higher tension [100]. Considering that running is predominantly eccentric, it is possible to evaluate muscle injury measuring the circulating levels of CK, a biomarker of stress and change in muscle activity in athletes [101, 102] and recreational athletes [103].

CK may denote a recent state of muscle deterioration due to physical exercise overloading. In this sense, the application of large loads of volume and/or intensity has led to considerable increases in the concentrations of CK circulating levels [100]. On the other hand, the taper period or recovery microcycles are the phases of training that provide significant muscle recovery and may promote a significant reduction in serum CK concentrations [104]. CK reduction was also seen in runners after 6 days [105]. These effects could possibly be due to the adaptation to the physical exercise during the training.

We observed that the adoption of microcycles of recovery in the Und-Und, Und-Lin, and Sta-Und groups showed a trend to reduce CK concentration in the serum of recreational athletes submitted to 8 weeks of running. This effect was mainly observed in the Und-Und group. Although the peak in the concentration of CK occurs after 24 h of exercise, it may extend for up to 72 h, being persistent. In high concentrations, CK is considered an important sign of chronic fatigue [102]. A reduction of around 6.09% of CK in runners was also observed after applying a week of step tapering off, when there is a sudden reduction in the training workload [105]. Others also observed an association between a trend to decrease CK levels and performance improvement in the taper period in runners [32] and swimmers [106].

We also verified that Und-Und group presented the highest trend to decline the circulating levels of LDH in recreational athletes after 8 weeks of running, as compared with the other training groups analyzed. On the other hand, the Sta-Lin group expressed a trend to increase LDH circulating levels. In this regard, the increase in the concentration of LDH in the blood may denote cell injury due to strenuous effort [8, 15]. These data allow us to propose that Und-Und model might be better tolerated and Sta-Lin program might promote more muscle injury. Our results showing a strong trend to decline CK and LDH circulating levels in recreational athletes after a specific taper-like period suggest that the Und-Und model might be more effective to prevent overtraining and stress due to oscillations between volume/intensity and recovery. Possibly, with a longer period of training, we could verify significant statistic changes in the circulating levels of CK and LDH enzymes in recreational athletes.

In relation to the circulating levels of AST and ALT, we verified that the Und-Und group showed the lowest trend to increase these parameters in the fourth and eighth weeks of training, when compared with the other groups. These changes can be explained by a possible effect of stimulus and recovery in the Und-Und model of periodization, i.e., taper-like model, which is the decrease of loads in the eighth week. In this condition, higher disponibility of ATP could contribute to maintain the cellular membrane integrity [3] and decrease exocytosis of AST and ALT to the circulation. Banister and colleagues also observed a decrease of AST besides CK and LDH in the taper period in athletes (32 days) [14]. Furthermore, a study with swimmers using two different taper periods showed a decrease in ALT and AST between the fourth and the ninth day [3].

The levels of AST and ALT significantly increased in the Sta-Lin group after 4 and 8 weeks of training. Skenderi et al. also verified increased gains in the circulating levels of AST and ALT in runners of long distance, and these effects were associated with an increase in the circulating levels of CK and LDH enzymes [107]. Additionally, high levels of AST and ALT were observed when runners of long duration performed extreme exercise [108]. It was verified a trend to increase ALT and a significant increase of AST in trained recreational athletes, after aerobic exercise [109]. In this context, possibly, the efflux of AST and ALT enzymes to blood circulation could be influenced by duration, type of exercise, intensity, and adaptation to physical training [3].

We evaluated the levels of U in our study because U as well as uric acid are formed during protein catabolism [110] and may indicate, indirectly, the state of proteolysis [111] and intensity monitoring, as described in exercise of endurance [2, 102, 112]. In addition, the verification of U levels may serve as a supplementary analysis of the anabolic/catabolic state of training [113] and as an indicator of muscle injury [14].

The levels of U showed a trend to increase in all groups evaluated in our study after 4 weeks of training. Excepting Sta-Lin group, all others, including Und-Und, Und-Lin, and Sta-Und groups, presented a trend to decline from the first to the eighth week. Similar to Und-Und and Und-Lin taper-like models, Coutts et al. observed a decrease in the circulating U levels in trained triathletes after a 2-week taper period, both in the normal and overload training groups [5].

The U level in the Sta-Lin group was significantly higher than in the Und-Und group after 8 weeks of training. In this regard, it is valid to point out that the U concentration can be increased after an intense effort. This alteration has been associated with a reduction in the renal blood flow, low glomerular filtration, and increased protein catabolism [114, 115]. In recreational athletes, after long-distance running, it is observed an increase in the serum U concentration and glomerular filtration in the first 24 h. After this period, while U concentration is still increased, the glomerular filtration decreases (*p* < 0.05) [116].

The taper period normally leads to an increase in the anabolism and a decrease in the catabolism, which can be verified by an increase in the FT levels and a decrease in the C levels, with performance improvement [5, 117]. All groups analyzed in our study presented a trend to increase C after 4 weeks of aerobic exercise. However, the levels of this hormone decreased in the Und-Und and Und-Lin groups in the taper-like period, after 8 weeks of training. Previous works with swimmers and triathletes showed a trend to decrease C levels associated with an increase in the performance after the taper period [5, 118]. The lower responses in C levels might be related to adaptation to the undulatory effects and load decrease in the taper-like period in the final week of training. In this context, other findings with cyclists and young rowers showed a trend to increase C levels with an increase in the performance after the taper period [119, 120]. The reduction of C levels can be due to a decrease in the protein catabolism favoring protein aggregation and reduction in their degradation [121]. In this case, it could be a mechanism of supercompensation during the taper-like period.

Differently, we verified that the Sta-Und and Sta-Lin groups showed an increase in the C levels after 8 weeks of training. Possibly, these groups did not present an efficient recovery, i.e., they did not have an anabolic response in the final week of training. Moreover, cortisol can promote an increase in the concentration of free fat acids and amino acids in the blood plasma when stimulating lipid catabolism and gluconeogenesis and synthesis of plasma and liver proteins [122], which is relevant for endurance athletes, for instance.

The Und-Und and Sta-Lin groups presented, respectively, the higher and lower trend to increase the levels of FT after eight weeks of training. Moreover, the Und-Lin and Sta-Und groups also showed an increase in the circulating testosterone (T) levels, even without the taper-like period in the final week of training. These data are in agreement with previous findings showing an increase in the plasma T concentration after 10 days of tapering in cyclists [123]. Although there was no load decrease in Sta-Und, this group showed an undulatory feature inside the weeks, the macrocycle. This effect on T levels might contribute for a stimulatory effect on recovery, which is characteristic of undulation load. In this context, trends to increase the circulating levels of total T and FT were related with the load intensity in the training [124], mainly in the taper period [32, 106, 118, 123], possibly due to the supercompensation recovery effects.

The FT/C ratio has been used as an important marker of training stress in different types of exercise [5, 9, 10, 32]. In our study, the FT/C ratio in recreational athletes of the Und-Und and Sta-Lin groups was significantly increased and decreased, respectively, after 8 weeks of training. In agreement with our findings in the Und-Und-group, Coutts and colleagues showed a trend to increase the FT/C ratio in triathletes [5], which could be explained by the metabolic scenarium in favor of the protein anabolism [125]. In relation to the opposite effect observed in the Sta-Lin group, with increased C and decreased T circulating levels, it could be explained by a catabolic effect during the aerobic training. In future studies, it would be interesting to evaluate the maximal lactate steady state which is considered a gold standard aerobic capacity marker and correlate through its threshold with VO_2max_. Moreover, analyses of oxidative stress markers, circulating levels of lipid peroxidation, as well as proinflammatory cytokines would help to better understand the physiological changes in the body of recreational athletes in response to modulations of distinct training loads. Conjointly, it might contribute to better understand the influence of trainings on muscle recovery and for physical educators and professionals to prescribe trainings to optimize physical adaptation of recreational athletes in running events.

## Conclusion

We verified that application of load reduction and taper-like period in the final week of short-term aerobic training interfere in the aerobic fitness of recreational athletes, being the effects related to changes in their general biological background. Our results indicate that recreational runners who perform physical exercises five times a week can increase their cardiorespiratory fitness, decrease muscle injuries, and increase FT/C ratio when they perform undulatory cycles weekly and monthly with decrease in volumes after 4 weeks of training. On the other hand, without taper-like periods, using staggered load manipulation, there is an increase in catabolism and muscle injury markers as well as a decreased FT/C ratio, thus indicating inefficient recovery.

## Abbreviations

ALT: Alanine aminotransferase
ANOVA: Analysis of variance
AST: Aspartate aminotransferase
BMI: Body mass index
C: Cortisol
CK: Creatine kinase
EDTA: Ethylenediaminetetraacetate
Hb: Hemoglobin
Ht: Hematocrit
LDH: Lactate dehydrogenase
MCHC: Mean corpuscular hemoglobin concentration
Sta-Lin: Staggered-linear
Sta-Und: Staggered-undulatory
T: Testosterone
T/C: Testosterone/cortisol ratio
U: Urea
Und-Lin: Undulatory-linear
Und-Und: Undulatory-undulatory
VE: Minute ventilation
V̇O_2max_: Maximal oxygen uptake
